# Ecological Niche Shifts Affect the Potential Invasive Risk of *Rapistrum rugosum* (L.) All. in China

**DOI:** 10.3389/fpls.2022.827497

**Published:** 2022-04-15

**Authors:** Xiaoqing Xian, Haoxiang Zhao, Rui Wang, Huijie Qiao, Jianyang Guo, Guifen Zhang, Wanxue Liu, Fanghao Wan

**Affiliations:** ^1^State Key Laboratory for Biology of Plant Diseases and Insect Pests, Institute of Plant Protection, Chinese Academy of Agricultural Sciences, Beijing, China; ^2^Institute of Zoology, Chinese Academy of Sciences, Beijing, China

**Keywords:** *Rapistrum rugosum*, ecological niche, suitable habitat, MaxEnt model, invasive risk

## Abstract

Ecological niche is a key concept that links species distributions. Ecological niche shifts are expected to affect the potential invasive risk of alien species. *Rapistrum rugosum* is an invasive agricultural weed in many countries. Wild populations of *R. rugosum* have been recorded in China, representing a great threat to the regional crops. Based on distribution records from different regions and relevant environmental variables, the present study predicted the potential distribution and estimated the invasive risk of *R. rugosum* in China. Ecological niche shifts strongly affected the potential invasive risk of *R. rugosum* in China. The two most important variables were annual temperature range (Bio7) and mean temperature of the coldest quarter (Bio11). The total suitable habitat for the species covered an area of 287.53 × 10^4^km^2^ and was mainly distributed in Southwest, Southeast, and Central China. Australia, Canada, Brazil, the United States, and Argentina accounted for over 90% of the inspection records of *R. rugosum* from Chinese entry ports during 2015–2018. The intercepted *R. rugosum* was frequently mixed in *Glycine max* (L.) Merr., *Hordeum vulgare* L., linseed, *Triticum aestivum* L., and *Sorghum bicolor* (L.) Moench. Moreover, 80% interceptions were recorded from Tianjin, Guangdong, Nanjing, and Chengdu customs. Climatic conditions do not limit the establishment capability of *R. rugosum* in China. Our results provide a theoretical reference for the development of monitoring and control measures for this invasive weed.

## Introduction

Biological invasions, as part of global change, are considered to be one of the important factors contributing to the decline in global biodiversity as well as high economic losses to the society (Mačić et al., 2018; Essl et al., 2020; Pyšek et al., 2020; Diagne et al., 2021; Pisani et al., 2021; Stinca et al., 2021). In recent years, China has become one of the countries that are most seriously affected by biological invasions worldwide (Yan et al., 2017). To date, more than 600 Invasive Alien Species (IAS) have been identified in China, of which more than 45% were invasive alien plants (Wan et al., 2017). *Rapistrum rugosum* (L.) All., a new reported invasive alien plant in China, belongs to the Brassicaceae family, and it is a relatively common weed in agricultural fields in wheat- and corn-growing regions (Pardo et al., 2019; Ali et al., 2020). *R. rugosum* is profusely branched and possesses a well-developed root system. In plots where wheat was mixed with *R. rugosum*, the former was at a competitive disadvantage, and its growth and yield were severely affected. In Australia, competition from *R. rugosum* led to 72–78% yield reduction in wheat (Manalil and Chauhan, 2019). *R. rugosum* can grow up to 1–5-feet-tall and bears a taproot that can become rather large (Lemke and Worthington, 1991). It can successfully outcompete native plant species, forming a vegetative cover of a single species (Manalil et al., 2018). *R. rugosum* originated in Central Europe, the Mediterranean, northern Africa, and western and temperate Asia (Brown, 1878), from where it has dispersed to the Americas, Oceania, and East Asia (Global Biodiversity Information Facility, 2021). The oval, dark brown, smooth, and minute seeds of *R. rugosum* can be dispersed over long distances aboard logs, other seeds, and contaminated materials. China has 306 international ports of entry (Cao, 2020). Information from different plant quarantine Customs revealed that over 100 interceptions of *R. rugosum* have been made each year since 2016. *R. rugosum* has been detected in the containers and seeds of incoming shipments to China.

Recently, wild populations of *R. rugosum* were discovered in China, representing a great potential threat to the regional crops. On Chinese mainland, *R. rugosum* was recorded for the first time in the Xi’an City (Shaanxi Province, northwestern China); it was found growing in patches and showed the tendency to disperse rapidly (Xun et al., 2020). Majority of the recent studies on *R. rugosum* mainly focused on its biological characteristics (Hichri et al., 2019), herbicide resistance (Hatami et al., 2016), and control measures (Simmons, 2005); however, only a few studies have assessed its global invasive risk in habitats through species distribution modeling. Risk assessment and early warning are the most effective strategies to prevent the introduction and dispersal of IAS (Greenberg et al., 2012). For IAS, a stable ecological niche is an invasive area that is identical to the region of origin (Liu et al., 2020). Meanwhile, a shifted ecological niche is different from the region of origin. However, it is impossible to accurately predict the distribution of IAS in the invasive area based on habitat information of its origin (Fernández and Hamilton, 2015). Although predictions of species distribution modeling are reliable in the model-fitted area, the model simulation capacity must be interpreted cautiously when switching to a new prediction area (Broennimann and Guisan, 2008; Beaumont et al., 2009; Tang et al., 2021). When IAS invade new habitats, they gradually adapt to the given conditions, expand their ecological niche, and adversely affect agroecosystems and biodiversity (Hejda et al., 2015). Thus, ecological niche models cannot predict ecological niches based solely on the information of species origin.

Species distribution models (SDMs) have been playing an increasingly important role in predicting the potential geographic distribution of species, particularly IAS (Srivastava et al., 2019; Thomas et al., 2021). The MaxEnt model uses species distribution records and the corresponding environmental variables in a given habitat, and this model is suitable for predicting the potential geographic distribution of species (Elith et al., 2011; Liu et al., 2017; Adhikari et al., 2019). In recent years, the application of the MaxEnt model has expanded not only to the examination of ecological degradation processes, such as biological invasions (Yeh et al., 2021) and ecological damage (Venne and Currie, 2021), but also to the potential risk assessment of IAS (Simpson and Prots, 2013). “kuenm,” an R package in the ecological niche model (ENM), uses MaxEnt as the modeling algorithm to automate the calibration of models, creation of optimized models and their transfer and evaluations, as well as assessment of extrapolation risks (Cobos et al., 2019). The MaxEnt model, combined with ArcGIS, has been widely used to identify areas at a high risk of IAS invasion (Kariyawasam et al., 2019) and to predict the impacts of climate change on IAS, enabling scientists and policymakers to establish effective and early warning strategies.

To this end, based on the optimized MaxEnt model, related environmental variables, and distribution records of *R. rugosum* in native, invasive, and native + invasive regions, the present study simulated the invasion risk habitats of *R. rugosum* in China. For the simulated risk area in China, we speculated that the ecological niche of *R. rugosum* would shift based on the distribution records of native, invasive, or native + invasive. Therefore, the risk area identified based on the native and invasive distribution records of *R. rugosum* was integrated as the final result of the invasion risk habitat in China. Further, the environmental variables that significantly affect the invasion risk habitats of *R. rugosum* in China were clarified. Finally, the specific distribution range in the invasion risk habitats of *R. rugosum* in China was explored to predict its dispersal risk and propose early warning measures.

## Materials and Methods

### Distribution Records of *Rapistrum rugosum*

**FIGURE 1:**
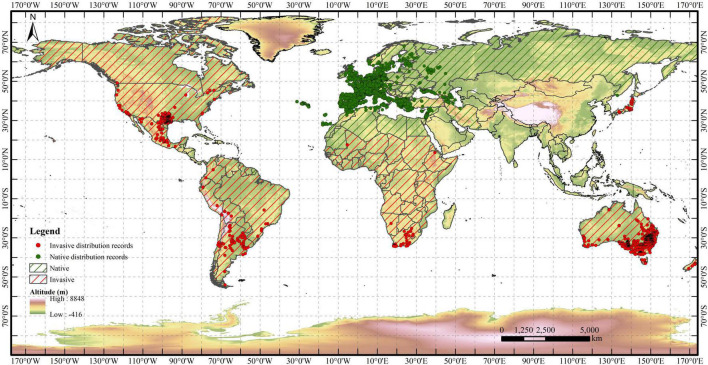
World distribution records of *Rapistrum rugosum.* Red and green points indicate invasive and native distribution records, respectively.

The distribution records of *R. rugosum* were collected from the Global Biodiversity Information Facility (GBIF^1^) and Invasive Species Compendium (ISC) of the Center for Agriculture and Bioscience International (CABI^2^). A total of 31.585 distribution records were obtained. Duplicate records and distribution points without detailed geographic information were removed using ENMtools (Warren et al., 2010). Regarding the resolution of the environmental variables, only one distribution point was retained within each 5 km × 5 km raster. Finally, a total of 8.938 valid distribution records of *R. rugosum* were retained. Among these, respectively, 6.259 and 2.679 distribution records were in native and invasive areas (Figure 1).

### Environmental Variables, Mapping, and Modeling

Raster files for 19 bioclimatic variables and elevation at a resolution of 2.5′ were downloaded from the World Climate Database Version 2.1^3^. This database includes detailed meteorological information from meteorological stations around the world during 1970–2000. Soil data were obtained from the Harmonized World Soil Database v1.2 (Fischer et al., 2008). Land use data for China in 2020 were downloaded from the Resource and Environment Science and Data Center^4^. All the data were converted to a resolution of 2.5′ (the same as that of the bioclimatic variables; Supplementary Table 1). The world administrative map was downloaded from the National Earth System Science Data Center, National Science and Technology Infrastructure of China^5^. MaxEnt 3.4.4 is freely available online^6^.

Correlation analysis of the 19 bioclimatic variables was performed using ENMtools to eliminate multivariate collinearity (Yang et al., 2013). The bioclimatic variables were selected through two steps: (1) the bioclimatic variables were imported into the MaxEnt model three times, and the bioclimatic variables with zero contribution were removed; and (2) all bioclimatic variables with contribution rates greater than zero were selected for correlation analysis using ENMtools. When the correlation coefficient of two bioclimatic variables was greater than or equal to 0.8 (Supplementary Figure 1), the one with the highest contribution rate was retained. The final environmental variables were retained for MaxEnt modeling (Supplementary Tables 2–4).

### MaxEnt Model Calibration

MaxEnt is an ecological niche model based on the theory of maximum entropy based on the Java platform (Phillips et al., 2006). The most important parameters of the MaxEnt model are the feature classes (FCs) and regularization multiplier (RM). FC and RM calibration can significantly improve the prediction accuracy of the MaxEnt model. In the present study, the MaxEnt model calibrated by setting different combinations of FCs and incremental RMs. FCs include five basic parameters, namely linear (L), quadratic (Q), product (P), threshold (T), and hinge (H), and there are 31 different combinations of FCs (Radosavljevic and Anderson, 2014). Generally, RM is set from 0.1 to 4 with an interval of 0.1. A total of 40 RM values were used in the present study. The “kuenm” package in R was used to create 1.240 candidate models. Finally, using R, significant models with the omission rate of >5% and delta Akaike Information Criterion (AICc) value of <2 (Cobos et al., 2019) were selected. The candidate model with the smallest delta AICc was selected for the final analysis.

### Model Settings and Evaluation

Following model calibration, 25% of the distribution records were used to test the MaxEnt model, and the remaining 75% were used to train the optimized MaxEnt model. In total, maximum 500 iterations and 10.000 background points were used. The importance of the environmental variables limiting *R. rugosum* distribution was assessed using the contribution rates and the Jackknife method. The receiver operating characteristic (ROC) curve and area under the curve (AUC) were used to test the accuracy of the model results. The ROC curve is an acceptance curve with the horizontal coordinate indicating the false positive rate (1 - specificity) and the vertical coordinate indicating the true positive rate (1 - omission rate) (Fan et al., 2006). Because the AUC values are not affected by the thresholds, it is an objective assessment of the model. An AUC value closer to 1 indicates that the model results are better. The evaluation criteria of model simulation accuracy were classified into three levels: poor (AUC ≤ 0.50), acceptable (0.5 < AUC ≤ 0.80), and excellent (0.80 < AUC ≤ 1.00) (Swets, 1988).

The maximum value of 10 replications was selected as the final MaxEnt model result in the present study. The ASCII raster layers were generated based on the logical value (P) of the presence probability of *R. rugosum*, ranging from 0 to 1. A higher *P* values indicates a higher probability of the presence of *R. rugosum*. The results were converted to a raster file and extracted using the administrative division map of China in ArcGIS. Then, the suitable habitats were ranked and visualized. The suitable areas were classified into four classes: highly suitable habitat (0.5 < *P* ≤ 1.0), moderately suitable habitat (0.3 < *P* ≤ 0.5), slightly suitable habitat (0.1 < *P* ≤ 0.3), and unsuitable habitat (0.0 ≤ *P* ≤ 0.1). Grids in each class were counted, and the proportion of suitable habitats in each class was calculated. Next, the precise invasion risk area of *R. rugosum* was determined by removing the suitable habitats of *R. rugosum* in water and unused land as the final result. The ecological niche overlap of *R. rugosum* was expressed in terms of the Schoener’s *D* (*D*) value in ENMtools. A higher Schoener’s *D* indicates a greater overlap of the ecological niches (Warren et al., 2010).

## Results

### Feature Classes and Regularization Multiplier of the Optimized Model

**FIGURE 2:**
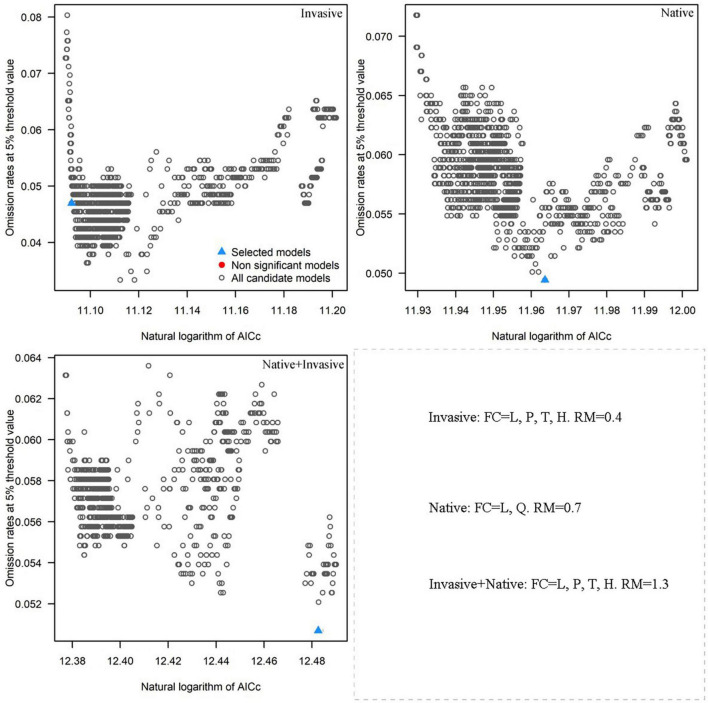
Omission rates and AICc values for all, non-significant, and selected “best” candidate models based on the invasive, native, and native + invasive records of *Rapistrum rugosum*.

The results of R analysis revealed that 1.160 of the 1.240 selected candidate models were statistically significant. The optimized model was selected based on the smallest delta AICc value. Based on the native distribution records of *R. rugosum*, the FCs were L and Q and the RM was 0.7 in the optimized model. Based on the invasive distribution records of *R. rugosum*, the FCs were L, P, T, and H and the RM was 0.4 in the optimized model. Based on the native + invasive distribution records of *R. rugosum*, the FCs were L, P, T, and H and the RM was 1.3 in the optimized model (Figure 2). Based on the native, invasive, and native + invasive distribution records of *R. rugosum*, the suitable habitats of *R. rugosum* were simulated using the MaxEnt model under current climatic conditions and the mean AUC values of, respectively, 0.813, 0.910, and 0.789 were obtained for the MaxEnt models Supplementary Figure 2). Model fitting based on the native or invasive distribution records of *R. rugosum* was excellent and that based on both native and invasive distribution records was acceptable.

### Significant Environmental Variables

**FIGURE 3:**
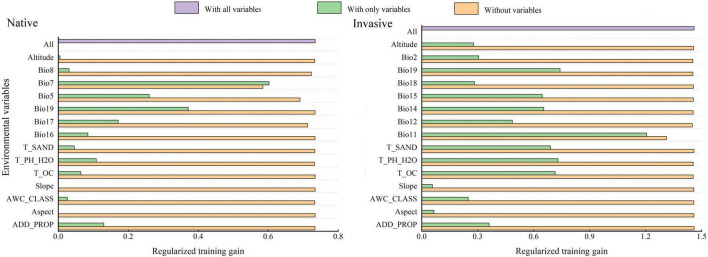
Regularized training gains of environmental variables using the Jackknife method in MaxEnt based on the native and invasive distribution records of *Rapistrum rugosum.*

Regularized training gain was used to determine the significant environmental variables, which were modeled in MaxEnt based on native and invasive distribution records of *R. rugosum.* Jackknife analysis revealed that the two most significant influencing factors for regularized training gain with a single variable were annual temperature range (Bio7) and mean temperature of the coldest quarter (Bio11). These two environmental variables provided information that the other environmental variables did not (Figure 3).

**FIGURE 4:**
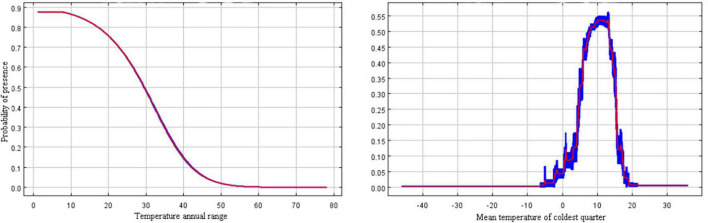
Response curves of the presence probability of *Rapistrum rugosum*.

In the present study, the relationship between the presence probability of *R. rugosum* and environmental variables was determined based on the response curves of environmental variables to the presence probability (Figure 4). When the presence probability of *R. rugosum* was greater than the threshold of highly suitable habitat classification (0.5), the corresponding interval was suitable for the growth of *R. rugosum.* The annual temperature range suitable for the growth of *R. rugosum* was 1.2–29.5°C, and the mean temperature of the coldest quarter suitable for the growth of *R. rugosum* was 8.1–13.4°C.

### Ecological Niche Shifts of *Rapistrum rugosum*

**FIGURE 5:**
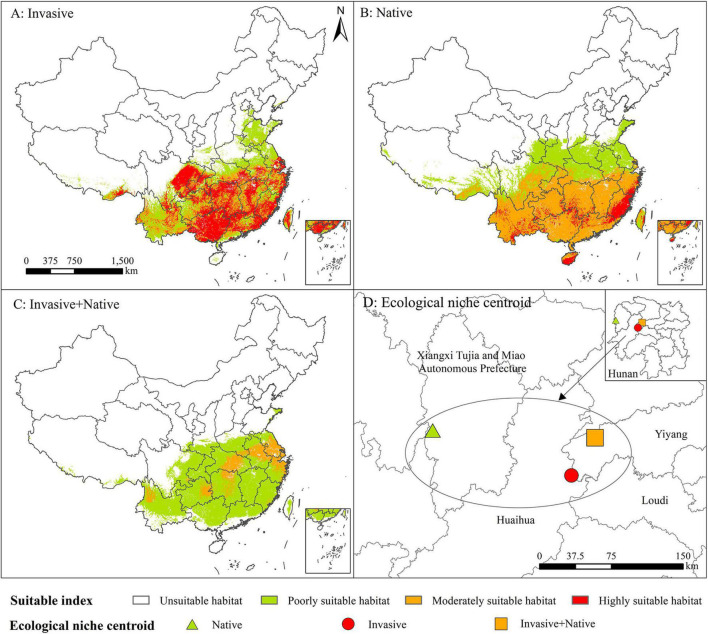
Potential geographic distribution of *Rapistrum rugosum* based on the native **(A)**, invasive **(B)**, and native + invasive **(C)** distribution records and the ecological niche centroid of *R. rugosum* **(D)**.

The suitable habitats and ecological niches of *R. rugosum* differed based on invasive, native, and native + invasive distribution records (Figure 5; Table 1 and Supplementary Table 5). Based on the invasive distribution records (Figure 5A), the area of the highly suitable habitat was 80.55 × 10^4^ km^2^, accounting for 8.39% of Chinese mainland, and these habitats were mainly distributed in southern and southeastern China. The area of the moderately suitable habitat was 60.35 × 10^4^ km^2^, accounting for 8.39% of Chinese mainland, and these habitats were mainly distributed around highly suitable habitats.

**TABLE 1:** Ecological niche overlap of *Rapistrum rugosum*.

Ecological niche overlap	Native + Invasive	Invasive	Native
Native + Invasive	1	0.92	0.25
Invasive	0.92	1	0.49
Native	0.25	0.49	1

Based on the native distribution records (Figure 5B), the area of the highly suitable habitat was 21.56 × 10^4^km^2^, accounting for 2.25% of Chinese mainland, and these habitats were mainly distributed in Zhejiang, Fujian, and Hainan provinces. The area of the moderately suitable habitat was 153.69 × 10^4^ km^2^, accounting for 16.01% of Chinese mainland, and these habitats were mainly distributed in Southwest, Southeast, and Central China.

Based on the native + invasive distribution records (Figure 5C), the area of the highly suitable habitat was 0.02 × 10^4^ km^2^, and these habitats were only distributed in Shanghai City. The area of the moderately suitable habitat was 31.33 × 10^4^ km^2^, accounting for 3.26% of Chinese mainland, and these habitats were mainly distributed in Jiangsu, Anhui, Hubei, Hunan, Guizhou, and Yunnan provinces.

The ecological niche centroid of *R. rugosum* was located in Hunan Province (Figure 5D). The highest ecological niche overlap (Schoener’s *D* = 0.92) was observed in the simulation results based on the invasive and native + invasive distribution records of *R. rugosum*, while the lowest overlap (Schoener’s *D* = 0.25) was between the native distribution records of *R. rugosum* (Table 1).

In summary, the results based on the invasive and native distribution records were consistent with the invasion risk habitats of *R. rugosum* in China, while the results based on the native + invasive distribution records were less consistent. Overall, the prediction of the risk habitats based on different distribution records revealed that the ecological niches of *R. rugosum* have shifted.

### Interception Records of *Rapistrum rugosum* at Chinese Ports

**FIGURE 6:**
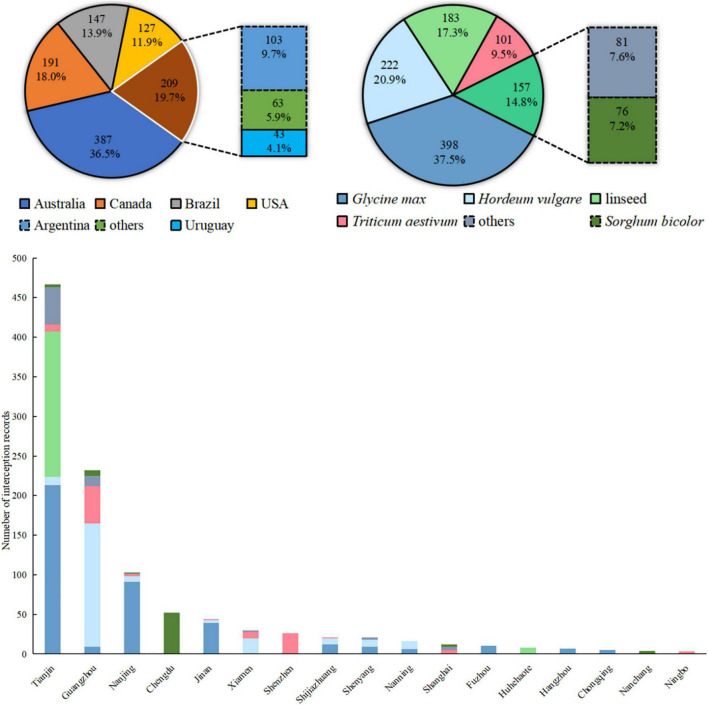
Imported commodities, countries of origin, and customs with interception records of *Rapistrum rugosum* in China during 2015–2018.

There have been 1.061 inspection records of *R. rugosum* from Chinese entry ports during 2015–2018 (Figure 6). The top five countries of origin associated with over 50 interceptions were Australia, Canada, Brazil, the United States, and Argentina, together accounting for >90% of all records. Of the *R. rugosum* interceptions recorded from imported commodities, 37.5% were from *Glycine max* (L.) Merr., 20.9% from *Hordeum vulgare* L., 17.3% from linseed, 9.5% from *Triticum aestivum* L., 7.2% from *Sorghum bicolor* (L.) Moench, and the remaining 7.6% from other goods. The frequent *R. rugosum* interceptions were recorded from 17 of 42 customs directly under the General Administration of Customs, China. Overall, 80% interception records were primarily from Tianjin, Guangdong, Nanjing, and Chengdu customs (more than 50 records each). Given the diversity of the countries of origin and commodities of *R. rugosum* interceptions at Chinese customs, the risk areas of this IAS in China must be first identified based on the invasive or native distribution records and then subjected to overlap analysis.

### Potential Invasive Risk Areas of *Rapistrum rugosum* in China

**FIGURE 7:**
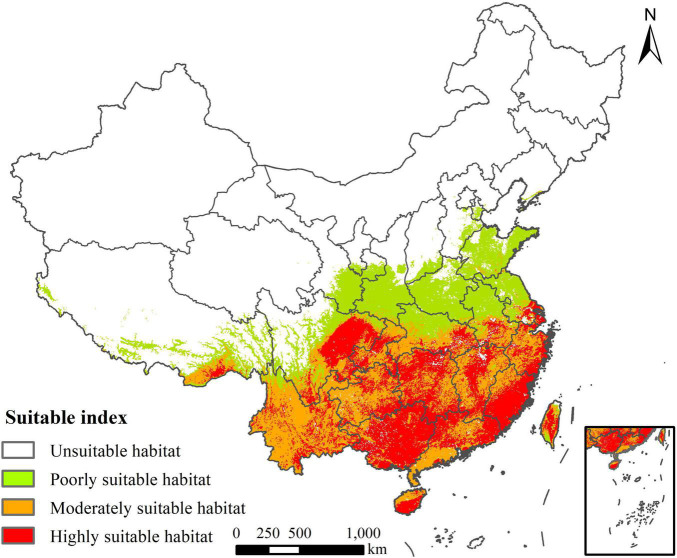
Potential invasive risk areas of *Rapistrum rugosum* in China.

The area of the highly suitable habitat of *R. rugosum* in China was 91.09 × 10^4^ km^2^, accounting for 9.49% of Chinese mainland, and these habitats were mainly distributed in Anhui, Zhejiang, Fujian, Jiangxi, Hunan, Guangdong, Hainan, Sichuan, Yunnan, Guizhou, Guangxi, Xizang, Shanghai, Chongqing, and Taiwan (Figure 7). The area of the moderately suitable habitat was 101.79 × 10^4^ km^2^, accounting for 10.6% of Chinese mainland, and these habitat was mainly distributed around the highly suitable habitat. The area of the total suitable habitat was 287.53 × 10^4^ km^2^, accounting for 29.95% of Chinese mainland.

## Discussion

### MaxEnt Model Development Using “kuenm”

Model calibration determines the combination of parameters that best represents the result by finding the best fit with the distribution records and environmental variables. In the present study, the R package “kuenm” was used to create optimized models based on model significance, performance, and simplicity. This approach helps prevent the overinterpretation of model outcomes (Cobos et al., 2019). “kuenm” has been previously used to optimize the MaxEnt model and predict the distribution of *Zanthoxylum bungeanum* in China; the optimized model reduced the overfitting degree, and the MaxEnt model fit was excellent (Zhuo et al., 2020). In the present study, the mean AUC values of the optimized MaxEnt models based on native, invasive, and native + invasive distribution records of *R. rugosum* were 0.813, 0.910, and 0.789, respectively, and the MaxEnt model fit was acceptable to excellent. Model optimization significantly improved the accuracy of the results.

### Ecological Niche Shifts for the Native and Invasive Populations of *Rapistrum rugosum*

Ecological niches play pivotal roles in understanding the patterns of species distribution (Liu et al., 2020). The native ecological niche of a species does not encompass all suitable habitats for its growth. The ecological niche of a species, particularly an IAS, is expected to shift if it is allowed to disperse freely (David and Menges, 2011). In 1785, *R. rugosum* was first reported in France, and thereafter, it rapidly spread over Europe and the Mediterranean region (Bruno and Solène, 2016). *R. rugosum* invaded the United States in 1883 and rapidly expanded throughout the Americas. Thus, it is considered a problematic agricultural weed worldwide. In the 2000s, *R. rugosum* was recorded from North and South America, southern Africa, East Asia, and Oceania (Staten Island Museum, 2021). In 2019, *R. rugosum* newly invaded northwestern China, without any information on its origin. The interception records indicated that the source countries of *R. rugosum* were home to both native and invasive populations of this weed. Invasive species can adapt to new environments in various ways and expand their ecological niches spatially, leading to inconsistencies between the ecological niches of the invasive and native populations (Zenni et al., 2014). The differences in phenology between the invasive and native populations of plants lead to a shift in the ecological niche of IAS (Wolkovich and Cleland, 2011). *R. rugosum* occupies widely variable climatic and ecological niches. The MaxEnt model cannot predict the potential invasive risk of IAS if they disperse beyond their native range (Sillero et al., 2021). Thus, ecological niche shifting should be considered when predicting the invasive risk habitats of IAS based on species distribution modeling.

Comprehensive multi-angle considerations are fundamental to predict the invasion risk areas of *R. rugosum* in China. Based on native, invasive, native + invasive distribution records of *R. rugosum*, our results showed that when modeled with the distribution records of native or invasive, the ecological niche had shifted. Ecological niche shifts during biological invasion have been proven in many case studies of IAS (Broennimann and Guisan, 2008; Manzoor et al., 2020). MaxEnt models consistently provided qualitatively different predictions based on native and invasive distribution records, mainly because SDMs fit on native range data poorly predict introduced range occupancy or because ecological niche shifts significantly reduce the transferability of MaxEnt SDMs (Atwater and Barney, 2021). Meanwhile, SDMs can only predict the initial spread of the introduced population but cannot accurately predict its future spread trend. When an IAS first invades a new habitat, the ecological niche does not change. However, the phenology, thermal tolerance, and life history of invasive plants may change after they gradually adapt to the new habitats and expand their ecological niche (Colautti et al., 2017; Atwater et al., 2018). Shifts in the realized niche are common during plant bioinvasion. Ecological niche shifts are practically important, as they alter the predicted geographical distribution of IAS (Pearman et al., 2008). Therefore, ecological niche shifts play a key role in predicting the spread of IAS. Studies on the identification of the invasive risk areas of IAS have mainly focused on a combination of native and invasive data, regardless of whether the species has spread to a broad ecological niche (Qin et al., 2015; Saranya et al., 2021). Our predictions of the invasive risk areas of *R. rugosum* based on specific climate variables together with a combination of native and invasive records may be less or more one-sided than predictions based on native and invasive records considered separately, which may have affected model accuracy. *R. rugosum* is a new IAS in China that has been intercepted from many countries (including native and invasive ranges). Therefore, multiple invasions of mixed populations are expected to contribute to the further spread of *R. rugosum* in China. Therefore, in the present study, the native and invasive records of *R. rugosum* were separately used to model the potential invasive risk areas of this IAS in China. Overall, the ecological niche of *R. rugosum* has shifted, affecting its potential invasion risk in China.

### Colonization and Dispersal Risk of *Rapistrum rugosum* in China and Significant Environmental Variables

*Rapistrum rugosum* is a globally important IAS. All customs in China should implement strict plant quarantine regulations on imported grain from Australia, Canada, Brazil, the United States, and Argentina to prevent the introduction of this species to new areas in the country. *R. rugosum* poses a great risk of invasion in China. The number of interception records from Tianjin Customs was the highest. Even though Tianjin customs is located in a slightly suitable habitat, it was also associated with the greatest risk of potential introduction. Moreover, Guangzhou Customs is associated with a high risk of potential introduction, because it is located in a highly suitable habitat and recorded many interceptions. Thus, Tianjin and Guangzhou customs should be closely monitored in terms of the quarantine of imported grain to prevent the introduction of *R. rugosum* mixed in imported grain. A wild population of *R. rugosum* has been discovered in Xi’an, and this population continues to spread in the surrounding areas. American countries accounted for over 50% of the total interception records of *R. rugosum*. In general, China, Europe, and North America have comparable climatic conditions. North America, Europe, and China have climate zones located south of the 40th parallel; therefore, most North American species can readily adapt to new habitats following their introduction into China and can effectively colonize in a relatively short time (Peel et al., 2007). According to the China Environment Report 2019, presented by the Ministry of Ecology and Environment, there are over 660 IAS in China; plants account for a majority of them, and over 50% originated from the United States. Therefore, climatic conditions cannot limit the ability of *R. rugosum* to establish populations in China.

Many Brassicaceae weeds germinate in a similar manner under different temperature conditions (Long et al., 2011). Temperature is an important variable affecting the germination of *R. rugosum* seeds (Ohadi et al., 2011; Hasanfard et al., 2021). Our analysis revealed that the most significant variables shaping *R. rugosum* distribution were annual temperature range (Bio7) and mean temperature of the coldest quarter (Bio11). These results further proved that temperature was an important factor limiting the survival of this weed. *R. rugosum* is a fast-growing weed, and its invasiveness is facilitated by its ability to germinate under a wide range of temperatures. In Australia, the seeds of *R. rugosum* could germinate at most temperatures ranging from 5 to 30°C (Ali et al., 2020). Our results showed that the annual temperature range and the mean temperature of the coldest quarter suitable for the growth of *R. rugosum* were 1.2–29.5°C and 8.1–13.4°C, respectively. We found that when the mean temperature of the coldest quarter was between 0 and 20°C, the survival probability of *R. rugosum* showed a fluctuating increase, followed by a fluctuating decrease. Our results are consistent with previous reports. Moreover, the mean annual temperature of suitable habitats for *R. rugosum* in China was >10°C, and the mean annual temperature of highly suitable habitats was >15°C. Overall, the mean annual temperature and mean temperature of the coldest quarter in southern China are suitable for the germination of *R. rugosum* seeds; thus, this region faces a risk of colonization and dispersal of *R. rugosum*.

In recent years, *R. rugosum* was frequently detected in the commodities and containers of importing grains and seeds, indicating that this weed can spread over long distances aboard other seeds, logs, and other contaminated materials. Early warning and control measures are essential to prevent and reduce *R. rugosum* invasion in China. Tianjin, Guangdong, Nanjing, and Chengdu customs have reported frequent interceptions of *R. rugosum*, underscoring the need for strict quarantine measures to prevent and reduce the introduction and survival of this IAS in the surrounding areas of entry ports and imported grain processing factories. Specific attention should be paid to the imported grains, including *Glycine max*, *Hordeum vulgare*, and linseed, from Australia, Canada, and Brazil. For wild populations of *R. rugosum*, chemical control tends to be problematic because of the potential risk of development of resistance to a number of specific herbicides (Hatami et al., 2016; Ntoanidou et al., 2019). Manual removal of the plant and its taproot and seed disposal are successful but time-consuming.

## Conclusion

The present study used the R package “kuenm” to develop comprehensive MaxEnt models. The overall model fit was excellent. Through the simulation of risk areas based on distribution records from different regions in China, we predicted that the ecological niche of *R. rugosum* would shift, and our model results confirmed this assumption. The two most significant variables shaping *R. rugosum* distribution were annual temperature range and mean temperature of the coldest quarter. Invasion risk assessment revealed that the area of the total suitable habitat for *R. rugosum* in China is 287.53 × 10^4^ km^2^, and these habitats are mainly distributed in Southwest, Southeast, and Central China. The potential habitats of *R. rugosum* accounted for a large proportion of Chinese mainland. Furthermore, climatic conditions will not limit the ability of *R. rugosum* to establish populations in China, and it has already successfully colonized specific regions within the country. Australia, Canada, Brazil, the United States, and Argentina are the five major source countries of *R. rugosum* in China. Meanwhile, *Glycine max*, *Hordeum vulgare*, linseed, *Triticum aestivum*, and *Sorghum bicolor* are the major grain sources of *R. rugosum*. Tianjin, Guangzhou, Nanjing, and Chengdu customs are the high-risk regions for the introduction of *R. rugosum* into China. Our results can serve as the reference to develop effective control measures against this IAS. Information about latitudinal clines in defense and joint clinical evolution of growth and defense in *R. rugosum* is essential for its adaptive evolution. Our further investigations will primarily focus on the re-establishment of heritable latitudinal clines in growth-related traits of *R. rugosum*.

## Data Availability Statement

The original contributions presented in the study are included in the article/Supplementary Material, further inquiries can be directed to the corresponding author.

## Author Contributions

XX, HZ, and WL: conception and design of the research. XX and HZ: acquisition of data, analysis and interpretation of data, statistical analysis, and drafting the manuscript. RW, HQ, JG, GZ, and FW: manuscript revision. All authors contributed to the article and approved the submitted version.

## Conflict of Interest

The authors declare that the research was conducted in the absence of any commercial or financial relationships that could be construed as a potential conflict of interest.

## Publisher’s Note

All claims expressed in this article are solely those of the authors and do not necessarily represent those of their affiliated organizations, or those of the publisher, the editors and the reviewers. Any product that may be evaluated in this article, or claim that may be made by its manufacturer, is not guaranteed or endorsed by the publisher.

## Footnotes

1
https://www.gbif.org/

2
https://www.cabi.org/isc

3
http://www.worldclim.org

4
https://www.resdc.cn/

5
http://www.geodata.cn

6
https://biodiversityinformatics.amnh.org/open_source/maxent/

## References

[B1] Adhikari P. Jeon J.-Y. Kim H. W. Shin M.-S. Adhikari P. Seo C. (2019). Potential impact of climate change on plant invasion in the Republic of Korea. *J. Ecol. Environ.* 43:36. 10.1186/s41610-019-0134-3

[B2] Ali H. H. Kebaso L. Manalil S. Chauhan B. S. (2020). Emergence and germination response of *Sonchus oleraceus* and *Rapistrum rugosum* to different temperatures and moisture stress regimes. *Plant Species Biol.* 35 16–23. 10.1111/1442-1984.12254

[B3] Atwater D. Z. Barney J. N. (2021). Climatic niche shifts in 815 introduced plant species affect their predicted distributions. *Glob. Ecol. Biogeogr.* 30 1671–1684. 10.1111/geb.13342

[B4] Atwater D. Z. Ervine C. Barney J. N. (2018). Climatic niche shifts are common in introduced plants. *Nat. Ecol. Evol.* 2 34–43. 10.1038/s41559-017-0396-z 29203919

[B5] Beaumont L. J. Gallagher R. V. Thuiller W. Downey P. O. Leishman M. R. Hughes L. (2009). Different climatic envelopes among invasive populations may lead to underestimations of current and future biological invasions. *Divers. Distrib.* 15 409–420. 10.1111/j.1472-4642.2008.00547.x

[B6] Broennimann O. Guisan A. (2008). Predicting current and future biological invasions: both native and invaded ranges matter. *Biol. Lett.* 4 585–589. 10.1098/rsbl.2008.0254 18664415 PMC2610080

[B7] Brown A. (1878). Plants introduced with ballast and on made land. *Bull. Torrey Bot. Club* 6 255–258. 10.2307/2476788

[B8] Bruno D. Solène R. (2016). *INPN - Données Flore Des CBN Agrégées Par La FCBN. Version 1.2. PatriNat, U. (OFB-CNRS-MNHN), Paris.* Paris: UMS PatriNat (OFB-CNRS-MNHN). 10.15468/omae84

[B9] Cao Z. D. (2020). *China’s Ports-Of-Entry 2019 Yearbook.* Beijing: China Customs Press.

[B10] Cobos M. E. Peterson A. T. Barve N. Osorio-Olvera L. (2019). Kuenm: an R package for detailed development of ecological niche models using Maxent. *PeerJ.* 7:e6281. 10.7717/peerj.6281 30755826 PMC6368831

[B11] Colautti R. I. Ågren J. Anderson J. T. (2017). Phenological shifts of native and invasive species under climate change: insights from the *Boechera–Lythrum* model. *Philos. Trans. R. Soc. Lond. B Biol. Sci.* 372:20160032. 10.1098/rstb.2016.0032 27920377 PMC5182428

[B12] David A. S. Menges E. S. (2011). Microhabitat preference constrains invasive spread of non-native natal grass (*Melinis repens*). *Biol. Invas.* 13 2309–2322. 10.1007/s10530-011-0044-5

[B13] Diagne C. Leroy B. Vaissière A. C. Gozlan R. E. Roiz D. Jarić I. (2021). High and rising economic costs of biological invasions worldwide. *Nature* 592 571–576. 10.1038/s41586-021-03405-6 33790468

[B14] Elith J. Phillips S. J. Hastie T. Dudík M. Chee Y. E. Yates C. J. (2011). A statistical explanation of MaxEnt for ecologists. *Divers. Distrib.* 17 43–57. 10.1111/j.1472-4642.2010.00725.x

[B15] Essl F. Lenzner B. Bacher S. Bailey S. Capinha C. Daehler C. (2020). Drivers of future alien species impacts: an expert-based assessment. *Glob. Change Biol.* 26 4880–4893. 10.1111/gcb.15199 32663906 PMC7496498

[B16] Fan J. Upadhye S. Worster A. (2006). Understanding receiver operating characteristic (ROC) curves. *Can. J. Emerg. Med.* 8 19–20. 10.1017/s1481803500013336 17175625

[B17] Fernández M. Hamilton H. (2015). Ecological niche transferability using invasive species as a case study. *PLoS One* 10:e0119891. 10.1371/journal.pone.0119891 25785858 PMC4364959

[B18] Fischer G. Nachtergaele F. Prieler S. Teixeira E. Tóth G. Velthuizen H. (2008). *Global Agro-ecological Zones Assessment for Agriculture (GAEZ 2008.* Laxenburg: FAO.

[B19] Global Biodiversity Information Facility (2021). *Rapistrum Rugosum* (L.) All. Available online at: https://www.gbif.org/species/5373368 (accessed December 22, 2021).

[B20] Greenberg M. Haas C. Cox A. Jr. Lowrie K. McComas K. North W. (2012). Ten most important accomplishments in risk analysis, 1980–2010. *Risk Anal.* 32 771–781. 10.1111/j.1539-6924.2012.01817.x 22548638 PMC7169135

[B21] Hasanfard A. Rastgoo M. Izadi Darbandi E. Nezami A. Chauhan B. S. (2021). Regeneration capacity after exposure to freezing in wild oat (*Avena ludoviciana* Durieu.) and turnipweed (*Rapistrum rugosum* (L.) All. in comparison with winter wheat. *Environ. Exp. Bot.* 181:104271. 10.1016/j.envexpbot.2020.104271

[B22] Hatami Z. M. Gherekhloo J. Rojano-Delgado A. M. Osuna M. D. Alcántara R. Fernández P. (2016). Multiple mechanisms increase levels of resistance in *Rapistrum rugosum* to ALS herbicides. *Front. Plant Sci.* 7:169. 10.3389/fpls.2016.00169 26941749 PMC4761845

[B23] Hejda M. Chytrý M. Pergl J. Pyšek P. (2015). Native-range habitats of invasive plants: are they similar to invaded-range habitats and do they differ according to the geographical direction of invasion? *Divers. Distrib.* 21 312–321. 10.1111/ddi.12269

[B24] Hichri A. O. Hichri F. Mastouri M. Brahmia A. Flamini G. Selmi B. (2019). Study of chemical composition, antibacterial and antioxidant activities of *Rapistrum rugosum* L. essential oils from flowers, leaves, and stems. *J. Essent. Oil Bear. Plants* 22 1416–1426. 10.1080/0972060X.2019.1682682

[B25] Kariyawasam C. S. Kumar L. Ratnayake S. S. (2019). Invasive plant species establishment and range dynamics in Sri Lanka under climate change. *Entropy* 21:571. 10.3390/e21060571 33267285 PMC7515060

[B26] Lemke D. E. Worthington R. D. (1991). *Brassica* and *Rapistrum* (Brassicaceae) in Texas. *Southwest. Nat.* 36 194–197. 10.2307/3671920

[B27] Liu C. Wolter C. Xian W. Jeschke J. M. (2020). Most invasive species largely conserve their climatic niche. *Proc. Natl Acad. Sci. U.S.A.* 117 23643–23651. 10.1073/pnas.2004289117 32883880 PMC7519298

[B28] Liu X. Liu H. Gong H. Lin Z. Lv S. (2017). Appling the one-class classification method of Maxent to detect an invasive plant *Spartina alterniflora* with time-series analysis. *Remote Sens.* 9:1120. 10.3390/rs9111120

[B29] Long R. L. Stevens J. C. Griffiths E. M. Adamek M. Gorecki M. J. Powles S. B. (2011). Seeds of *Brassicaceae* weeds have an inherent or inducible response to the germination stimulant karrikinolide. *Ann. Bot.* 108 933–944. 10.1093/aob/mcr198 21821831 PMC3177676

[B30] Mačić V. Albano P. G. Almpanidou V. Claudet J. Corrales X. Essl F. (2018). Biological invasions in conservation planning: a global systematic review. *Front. Mar. Sci.* 5:178. 10.3389/fmars.2018.00178

[B31] Manalil S. Chauhan B. S. (2019). Interference of turnipweed (*Rapistrum rugosum*) and Mexican pricklepoppy (*Argemone mexicana*) in wheat. *Weed Sci.* 67 666–672. 10.1017/wsc.2019.42

[B32] Manalil S. Haider Ali H. Chauhan B. S. (2018). Germination ecology of turnip weed (*Rapistrum rugosum* (L.) All.) in the northern regions of Australia. *PLoS One* 13:e0201023. 10.1371/journal.pone.0201023 30024963 PMC6053197

[B33] Manzoor S. A. Griffiths G. Obiakara M. C. Esparza-Estrada C. E. Lukac M. (2020). Evidence of ecological niche shift in *Rhododendron ponticum* (L.) in Britain: hybridization as a possible cause of rapid niche expansion. *Ecol. Evol.* 10 2040–2050. 10.1002/ece3.6036 32128136 PMC7042765

[B34] Ntoanidou S. Madesis P. Eleftherohorinos I. (2019). Resistance of *Rapistrum rugosum* to tribenuron and imazamox due to Trp574 or Pro197 substitution in the acetolactate synthase. *Pestic. Biochem. Physiol.* 154 1–6. 10.1016/j.pestbp.2018.12.001 30765051

[B35] Ohadi S. Mashhadi H. R. Tavakol-Afshari R. (2011). Effects of storage and burial on germination responses of encapsulated and naked seeds of turnipweed (*Rapistrum rugosum*) to light. *Weed Sci.* 59 483–488. 10.1614/WS-D-10-00153.1

[B36] Pardo G. Marí A. I. Aibar J. Vilaplana L. Cirujeda A. (2019). Bastard cabbage (*Rapistrum rugosum* L.) resistance to Tribenuron-methyl and Iodosulfuron-methyl-sodium in Spain and alternative herbicides for its control. *Agronomy* 9:492. 10.3390/agronomy9090492

[B37] Pearman P. B. Guisan A. Broennimann O. Randin C. F. (2008). Niche dynamics in space and time. *Trends Ecol. Evol.* 23 149–158. 10.1016/j.tree.2007.11.005 18289716

[B38] Peel M. C. Finlayson B. L. McMahon T. A. (2007). Updated world map of the Köppen-Geiger climate classification. *Hydrol. Earth Syst. Sci.* 11 1633–1644. 10.5194/hess-11-1633-2007

[B39] Phillips S. J. Anderson R. P. Schapire R. E. (2006). Maximum entropy modeling of species geographic distributions. *Ecol. Modell.* 190 231–259. 10.1016/j.ecolmodel.2005.03.026

[B40] Pisani D. Pazienza P. Perrino E. V. Caporale D. De Lucia C. (2021). The economic valuation of ecosystem services of biodiversity components in protected areas: a review for a framework of analysis for the gargano national park. *Sustainability* 13:11726. 10.3390/su132111726

[B41] Pyšek P. Hulme P. E. Simberloff D. Bacher S. Blackburn T. M. Carlton J. T. (2020). Scientists’ warning on invasive alien species. *Biol. Rev. Camb. Philos. Soc.* 95 1511–1534. 10.1111/brv.12627 32588508 PMC7687187

[B42] Qin Z. Zhang J. E. DiTommaso A. Wang R. L. Wu R. S. (2015). Predicting invasions of *Wedelia trilobata* (L.) Hitchc. with Maxent and GARP models. *J. Plant Res.* 128 763–775. 10.1007/s10265-015-0738-3 26045231

[B43] Radosavljevic A. Anderson R. P. (2014). Making better Maxent models of species distributions: complexity, overfitting and evaluation. *J. Biogeogr.* 41 629–643. 10.1111/jbi.12227

[B44] Saranya K. R. L. Lakshmi T. V. Reddy C. S. (2021). Predicting the potential sites of *Chromolaena odorata* and *Lantana camara* in forest landscape of Eastern Ghats using habitat suitability models. *Ecol. Inform* 66:101455. 10.1016/j.ecoinf.2021.101455

[B45] Sillero N. Arenas-Castro S. Enriquez-Urzelai U. Vale C. G. Sousa-Guedes D. Martínez-Freiría F. (2021). Want to model a species niche? A step-by-step guideline on correlative ecological niche modelling. *Ecol. Modell.* 456:109671. 10.1016/j.ecolmodel.2021.109671

[B46] Simmons M. T. (2005). Bullying the bullies: the selective control of an exotic, invasive annual (*Rapistrum rugosum*) by oversowing with a competitive native species (*Gaillardia pulchella*). *Restor. Ecol.* 13 609–615. 10.1111/j.1526-100X.2005.00078.x

[B47] Simpson M. Prots B. (2013). Predicting the distribution of invasive plants in the Ukrainian Carpathians under climatic change and intensification of anthropogenic disturbances: implications for biodiversity conservation. *Environ. Conserv.* 40 167–181. 10.1017/S037689291200032X

[B48] Srivastava V. Lafond V. Griess V. C. (2019). Species distribution models (SDM): applications, benefits and challenges in invasive species management. *CAB Rev.* 14 1–13. 10.1079/PAVSNNR201914020

[B49] Staten Island Museum (2021). *Staten Island museum. Occurrence Dataset.* Staten Island, NY: Staten Island Museum. 10.15468/ctqpb5

[B50] Stinca A. Musarella C. M. Rosati L. Laface V. L. A. Licht W. Fanfarillo E. (2021). Italian vascular flora: new findings, updates and exploration of floristic similarities between regions. *Diversity* 13:600. 10.3390/d13110600

[B51] Swets J. A. (1988). Measuring the accuracy of diagnostic systems. *Science* 240 1285–1293. 10.1126/science.3287615 3287615

[B52] Tang X. Yuan Y. Liu X. Zhang J. (2021). Potential range expansion and niche shift of the invasive *Hyphantria cunea* between native and invasive countries. *Ecol. Entomol.* 46 910–925. 10.1111/een.13028

[B53] Thomas S. M. Verhoeven M. R. Walsh J. R. Larkin D. J. Hansen G. J. A. (2021). Species distribution models for invasive *Eurasian watermilfoil* highlight the importance of data quality and limitations of discrimination accuracy metrics. *Ecol. Evol.* 11 12567–12582. 10.1002/ece3.8002 34594521 PMC8462136

[B54] Venne S. Currie D. J. (2021). Can habitat suitability estimated from MaxEnt predict colonizations and extinctions? *Divers. Distrib.* 27 873–886. 10.1111/ddi.13238

[B55] Wan F. Jiang M. Zhan A. (2017). “Biological invasions and its management in China,” in *Biological Invasion and Its Research in China: An Overview*, Vol. 1 eds Yan Y. Xian X. Jiang M. Wang F. (Dordrecht: Springer), 3–20. 10.1007/978-94-024-0948-2

[B56] Warren D. L. Glor R. E. Turelli M. (2010). ENMTools: a toolbox for comparative studies of environmental niche models. *Ecography* 33 607–611. 10.1111/j.1600-0587.2009.06142.x

[B57] Wolkovich E. M. Cleland E. E. (2011). The phenology of plant invasions: a community ecology perspective. *Front. Ecol. Environ.* 9 287–294. 10.1890/100033

[B58] Xun L. Li S. Li W. Zhou Y. Lu Y. Mao S. (2020). Newly recorded plants of Brassicaceae from Shaanxi Province. *Acta Bot. Boreali Occidentalia Sin.* 40 1425–1435. 10.7606/.j.issn.100-4025.2020.05.1428

[B59] Yan Y. Xian X. Jiang M. Wan F. (2017). “Biological invasion and its research in China: an overview,” in *Biological Invasions And Its Management In China: 2*, eds Wan F. Jiang M. Zhan A. (Dordrecht: Springer Netherlands), 3–19. 10.1371/journal.pone.0001208

[B60] Yang X. Kushwaha S. P. S. Saran S. Xu J. Roy P. S. (2013). Maxent modeling for predicting the potential distribution of medicinal plant. *Justicia adhatoda L*. in lesser himalayan foothills. *Ecol. Eng.* 51 83–87. 10.1016/j.ecoleng.2012.12.004

[B61] Yeh H. T. Cheah H. Y. Chiu M. C. Liao J. R. Ko C. C. (2021). Assessment of potential invasion for six phytophagous quarantine pests in Taiwan. *Sci. Rep.* 11:10666. 10.1038/s41598-021-89914-w 34021194 PMC8140104

[B62] Zenni R. D. Bailey J. K. Simberloff D. (2014). Rapid evolution and range expansion of an invasive plant are driven by provenance–environment interactions. *Ecol. Lett.* 17 727–735. 10.1111/ele.12278 24703489

[B63] Zhuo Z. Xu D. Pu B. Wang R. Ye M. (2020). Predicting distribution of *Zanthoxylum bungeanum* Maxim. in China. *BMC Ecol.* 20:46. 10.1186/s12898-020-00314-6 32782004 PMC7422582

