# Supplementary material for: Ecological Niche Shifts Affect the Potential Invasive Risk of Rapistrum rugosum (L.) All. in China
Source: Front Plant Sci. 2022 Apr 15;13:827497. doi: 10.3389/fpls.2022.827497 (PMC9051486; doi:10.3389/fpls.2022.827497)
Supplement: Supplementary file 1 [file Data_Sheet_1.docx]

Supplementary Material

# Supplementary Figures and Tables

## Supplementary Figures


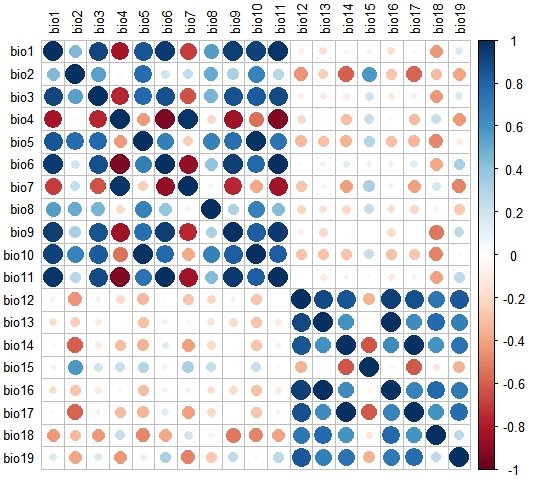


**Supplementary Figure S1** Pearson correlation coefficients for the 19 bioclimatic variables

**
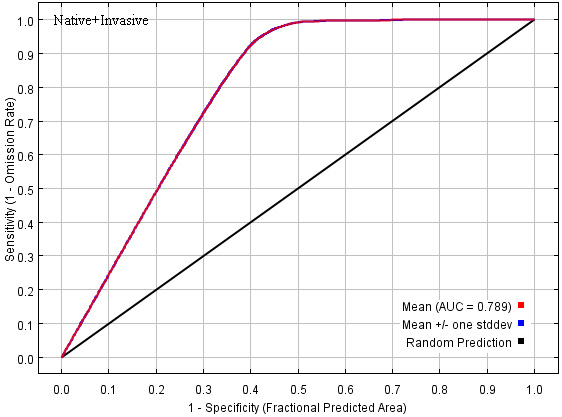

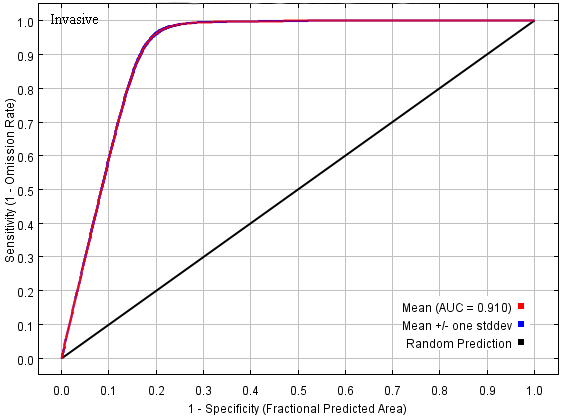
**

**
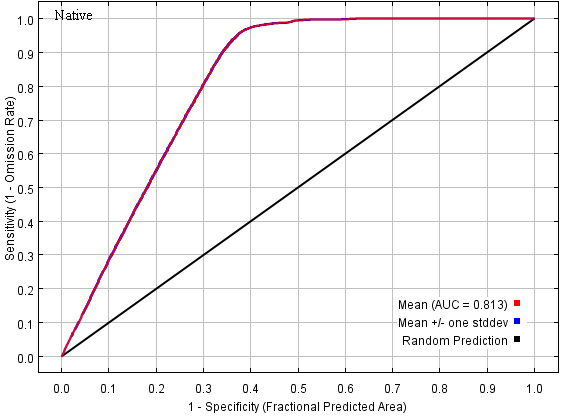
**

**Supplementary Figure S2** Receiver operating characteristic curve of *Rapistrum rugosum*

## Supplementary Tables

**Supplementary Table S1** All environmental variables

| Variables | Description | Unit |
| --- | --- | --- |
| Bio1 | Annual mean temperature | ℃ |
| Bio2 | Mean diurnal range | ℃ |
| Bio3 | Isothermality | - |
| Bio4 | Temperature seasonality | ℃ |
| Bio5 | Max temperature of warmest month | ℃ |
| Bio6 | Min temperature of coldest month | ℃ |
| Bio7 | Temperature annual range | ℃ |
| Bio8 | Mean temperature of wettest quarter | ℃ |
| Bio9 | Mean temperature of driest quarter | ℃ |
| Bio10 | Mean temperature of warmest quarter | ℃ |
| Bio11 | Mean temperature of coldest quarter | ℃ |
| Bio12 | Annual precipitation | mm |
| Bio13 | Precipitation of wettest month | mm |
| Bio14 | Precipitation of driest month | mm |
| Bio15 | Precipitation seasonality | - |
| Bio16 | Precipitation of wettest quarter | mm |
| Bio17 | Precipitation of driest quarter | mm |
| Bio18 | Precipitation of warmest quarter | mm |
| Bio19 | Precipitation of coldest quarter | mm |
| Altitude | Altitude | m |
| Slope | Slope | ° |
| Aspect | Aspect | - |
| T_PH_H_2_O | Topsoil pH (H_2_O) | -log(H^+^) |
| T_SAND | Topsoil Sand Fraction | % wt. |
| ADD_PROP | Other properties (gelic, vertic, petric) | Code |
| AWC_CLASS | AWC range | Code |
| T_OC | Topsoil Organic Carbon | % weight |

**Supplementary Table S2** Environmental variables related to the distribution of *Rapistrum rugosum* based on invasive distribution records

| Variables | Description | Unit |
| --- | --- | --- |
| Bio2 | Mean diurnal range | ℃ |
| Bio11 | Mean temperature of coldest quarter | ℃ |
| Bio12 | Annual precipitation | mm |
| Bio14 | Precipitation of driest month | mm |
| Bio15 | Precipitation seasonality | - |
| Bio18 | Precipitation of warmest quarter | mm |
| Bio19 | Precipitation of coldest quarter | mm |
| Altitude | Altitude | m |
| Slope | Slope | ° |
| Aspect | Aspect | - |
| T_PH_H_2_O | Topsoil pH (H_2_O) | -log(H^+^) |
| T_SAND | Topsoil Sand Fraction | % wt. |
| ADD_PROP | Other properties (gelic, vertic, petric) | Code |
| AWC_CLASS | AWC range | Code |
| T_OC | Topsoil Organic Carbon | % weight |

**Supplementary Table S3** Environmental variables related to the distribution of *Rapistrum rugosum* based on native distribution records

| Variables | Description | Unit |
| --- | --- | --- |
| Bio5 | Max temperature of warmest month | ℃ |
| Bio7 | Temperature annual range | ℃ |
| Bio8 | Mean temperature of wettest quarter | ℃ |
| Bio16 | Precipitation of wettest quarter | mm |
| Bio17 | Precipitation of driest quarter | mm |
| Bio19 | Precipitation of coldest quarter | mm |
| Altitude | Altitude | m |
| Slope | Slope | ° |
| Aspect | Aspect | - |
| T_PH_H_2_O | Topsoil pH (H_2_O) | -log(H^+^) |
| T_SAND | Topsoil Sand Fraction | % wt. |
| ADD_PROP | Other properties (gelic, vertic, petric) | Code |
| AWC_CLASS | AWC range | Code |
| T_OC | Topsoil Organic Carbon | % weight |

**Supplementary Table S4** Environmental variables related to the distribution of *Rapistrum rugosum* based on native + invasive distribution records

| Variables | Description | Unit |
| --- | --- | --- |
| Bio2 | Mean diurnal range | ℃ |
| Bio6 | Min temperature of coldest month | ℃ |
| Bio7 | Temperature annual range | ℃ |
| Bio12 | Annual precipitation | mm |
| Bio14 | Precipitation of driest month | mm |
| Bio16 | Precipitation of wettest quarter | mm |
| Bio19 | Precipitation of coldest quarter | mm |
| Altitude | Altitude | m |
| Slope | Slope | ° |
| Aspect | Aspect | - |
| T_PH_H_2_O | Topsoil pH (H_2_O) | -log(H^+^) |
| T_SAND | Topsoil Sand Fraction | % wt. |
| ADD_PROP | Other properties (gelic, vertic, petric) | Code |
| AWC_CLASS | AWC range | Code |
| T_OC | Topsoil Organic Carbon | % weight |

**Supplementary Table S5** On the basis of native, invasive and native+invasive distribution records, the suitable habitat areas of *Rapistrum rugosum* under current climatic (10^4^ km^2^)

| Region | Highly suitable habitat | moderately suitable habitat | poorly suitable habitat | Total suitable habitat |
| --- | --- | --- | --- | --- |
| Invasive | 80.55 | 60.35 | 75.78 | 216.68 |
| Native | 21.56 | 153.69 | 101.06 | 276.31 |
| Invasive+Native | 0.02 | 31.33 | 166.92 | 198.27 |
